# Modeling and forecasting the spread tendency of the COVID-19 in China

**DOI:** 10.1186/s13662-020-02940-2

**Published:** 2020-09-14

**Authors:** Deshun Sun, Li Duan, Jianyi Xiong, Daping Wang

**Affiliations:** 1 Shenzhen Key Laboratory of Tissue Engineering, Shenzhen Laboratory of Digital Orthopedic Engineering, Guangdong Provincial Research Center for Artificial Intelligence and Digital Orthopedic Technology, Shenzhen Second People’s Hospital (The First Hospital Affiliated to Shenzhen University, Health Science Center), Shenzhen, 518035 P.R. China; 2 Department of Biomedical Engineering, Southern University of Science and Technology, Shenzhen, 518055 P.R. China

**Keywords:** COVID-19, Mathematical modeling, Parameter estimation, Forecasting, Control strategy

## Abstract

To forecast the spread tendency of the COVID-19 in China and provide effective strategies to prevent the disease, an improved SEIR model was established. The parameters of our model were estimated based on collected data that were issued by the National Health Commission of China (NHCC) from January 10 to March 3. The model was used to forecast the spread tendency of the disease. The key factors influencing the epidemic were explored through modulation of the parameters, including the removal rate, the average number of the infected contacting the susceptible per day and the average number of the exposed contacting the susceptible per day. The correlation of the infected is 99.9% between established model data in this study and issued data by NHCC from January 10 to February 15. The correlation of the removed, the death and the cured are 99.8%, 99.8% and 99.6%, respectively. The average forecasting error rates of the infected, the removed, the death and the cured are 0.78%, 0.75%, 0.35% and 0.83%, respectively, from February 16 to March 3. The peak time of the epidemic forecast by our established model coincided with the issued data by NHCC. Therefore, our study established a mathematical model with high accuracy. The aforementioned parameters significantly affected the trend of the epidemic, suggesting that the exposed and the infected population should be strictly isolated. If the removal rate increases to 0.12, the epidemic will come to an end on May 25. In conclusion, the proposed mathematical model accurately forecast the spread tendency of COVID-19 in China and the model can be applied for other countries with appropriate modifications.

## Background

COVID-19 is an emergent infectious disease caused by the novel coronavirus 2019-nCoV. It was first detected in Wuhan, China in December of 2019 [1, 2]. From the COVID-19 outbreak in Wuhan, it has spread across China and beyond. By March 3, 2020, 80270 infection cases have been confirmed and 2981 death cases have been reported. Patients with confirmed COVID-19 are the main sources of infection, and asymptomatic patients are also potential sources of 2019-nCoV infection. Respiratory droplets and contact transmission are considered to be the most important routes of transmission of 2019-nCoV. The population is generally susceptible to the infection disease [3], and the elderly and those with chronic diseases are most susceptible to severe forms of COVID-19 [4, 5]. Multiple strategies have been developed to fight against the spread of COVID-19, including strict isolation, early diagnosis and supporting treatment. Currently, there is no specific medicine to combat the novel coronavirus. Therefore, it is a crucial step to forecast the spread tendency of the acute infectious disease based on the epidemiology data. Mathematical modeling is one of the most effective methods for forecasting of infectious disease outbreaks and thus yield valuable insights suggest how future efforts may be improved. An important method for epidemiological studies of such acute infectious diseases is mathematical modeling [6, 7].

Since the epidemic outbreak, some scholars have established an ordinary differential equation model to forecast the spread of COVID-19 [8–19]. Wu *et al*. [12] calculated that the basic reproductive number of new pneumonia ($R_{0}$) is 2.68 based on the established susceptible–exposed–infected–removed (SEIR) model, and forecast that the number of infected people in Wuhan would be 75,815 on January 25, 2020. Moreover, the authors also forecast the number of infected people imported from Wuhan to Chongqing, Beijing, Shanghai, Guangzhou and Shenzhen. However, the number of infections reported in this study is inconsistent with the number issued by NHCC (1870 cases), and the difference is large. Zhou *et al*. [14] used the SEIR compartment model to characterize the early spreading of COVID-19, and forecast basic reproduction number ($R_{0}$) is between 2.2 and 3.0. However, the model has not considered that the susceptible can be infected by confirmed patients during the incubation period that cannot be ignored. Besides, Tang *et al*. [8] established a complicated model and forecast that the control reproduction number ($R_{0}$) may be as high as 6.47 (95% CI 5.71–7.23). Using their estimated parameter values, the number of the infected will reach the peak around March 10, 2020 and the peak number of the infected is $1.63 \times 10^{5}$. However, the peak number issued by NHCC is 58,016 and the time of reaching the peak is February 17. Therefore, a more accurate mathematical model is highly anticipated to forecast the spread tendency of COVID-19.

In this study, we re-established a SEIR model of COVID-19 based on its transmission mechanism. The aim of this study was to obtain a more accurate mathematical model to forecast the spread tendency of epidemic and provide guidance to control the spread of COVID-19.

## Methods and data

### Theoretical mathematical model

The most classical model for studying infectious diseases is the SIR compartment model, which was proposed by Kermack and McKendrick in 1927 when studying the black plague [20]. SIR model divides population into three categories: Susceptible, which means the uninfected persons who lack immunity; Infective, which means the persons who are capable of spreading the disease to a susceptible person; removed, which means the dead or healed [21, 22]. The basic model is as follows:

1 $$ \begin{gathered} \frac{ds(t)}{dt} = - \beta i(t)s(t), \\ \frac{di(t)}{dt} = \beta i(t)s(t) - \gamma i(t), \\ \frac{dr}{dt} = \gamma i(t). \end{gathered} $$

Here $s(t)$, $i(t)$ and $r(t)$ are the susceptible, the infected and the removed, respectively. *β* is infection rate and *γ* is removal rate.

Gradually, researchers realized that the incubation period, the time between exposure and the start of symptoms, should be taken into consideration [23–26]. Therefore, the classic SEIR model was modified as follows:

2 $$ \begin{gathered} \frac{dS(t)}{dt} = \lambda- \beta S(t)I(t) - dS(t), \\ \frac{dE}{dt} = \beta S(t)I(t) - (d + \alpha)E(t), \\ \frac{dI}{dt} = \alpha E(t) - (d + \gamma+ \delta)I(t), \\ \frac{dR}{dt} = \gamma I(t) - dR(t). \end{gathered} $$

**Figure 1:**
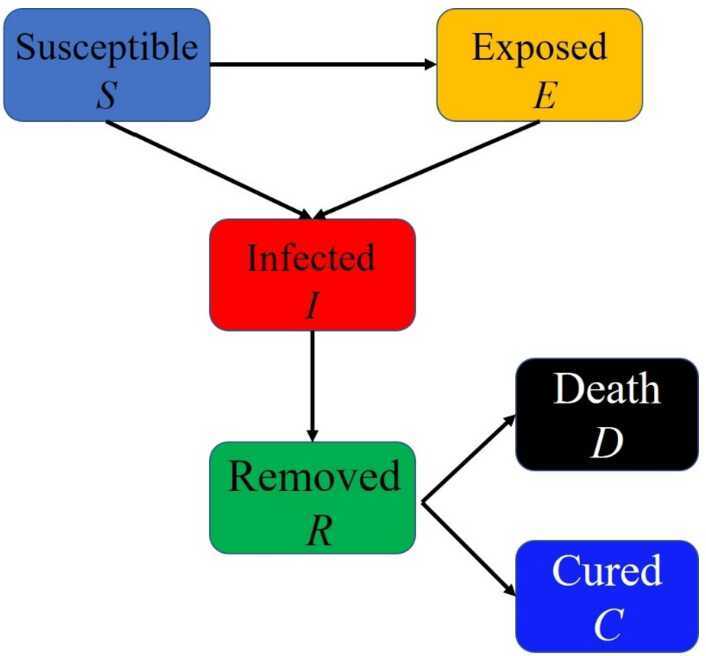
The transmission mechanism of COVID-19

According to the classic SEIR model, this study divided the population into four groups: susceptible (*S*), exposed (*E*), infected (*I*) and removed (*R*). As the susceptible can be infected by the exposed and COVID-19 confirmed infection, we proposed the following transmission mode (Fig. 1).

In this epidemic, the natural mortality of newborns and the natural death of all population were ignored for a short period of time. Because the susceptible can be infected by the exposed and the infected, we established the following mathematical model in this study:

3 $$\begin{aligned}& \frac{dS(t)}{dt} = - \frac{r\beta S(t)I(t)}{N(t)} - \frac{r_{2}\beta_{2}S(t)E(t)}{N(t)}, \\& \frac{dE(t)}{dt} = \frac{r\beta S(t)I(t)}{N(t)} + \frac{r_{2}\beta_{2}S(t)E(t)}{N(t)} - \delta E(t), \\& \frac{dI(t)}{dt} = \delta E(t) - \gamma I(t), \\& \frac{dR(t)}{dt} = \gamma I(t), \\& \frac{dD(t)}{dt} = \gamma_{0}I(t), \\& C(t) = R(t) - D(t), \\& N(t) = S(t) + E(t) + I(t) + R(t). \end{aligned}$$

Here $S(t)$, $E(t)$, $I(t)$, $R(t)$, $D(t)$ and $C(t)$ are the susceptible, the exposed (latently infected), the infected, the removed, the death and the cured, respectively. *r* represents the average number of the infected contacting the susceptible per day, *β* represents the probability of susceptible person being infected by infected person. Therefore, the infect rate of the susceptible by infected person is $\frac{r\beta S(t)I(t)}{N(t)}$. However, the susceptible can also be infected by the exposed, we use parameter $r_{2}$ to represent the average number of the exposed contacting the susceptible per day, $\beta_{2}$ represents the probability of susceptible person being infected by exposed person. The infect rate of the susceptible by infected person is $\frac{r_{2}\beta_{2}S(t)E(t)}{N(t)}$. *δ* is the probability of the exposed to become the infected. *γ* is the removal rate which includes the cure rate and death rate. $\gamma_{0}$ is the death rate due to COVID-19 disease. $C(t) = R(t) - D(t)$. $N(t)$ is the total population and $N(t) = S(t) + E(t) + I(t) + R(t)$.

### Data

Next, we estimated the parameters based on the number of the infected, the cured and deaths that issued by NHCC every day at 12 pm to obtain an accurate model. Based on the data issued by the NHCC from January 10, 2020 to March 3, 2020, the statistical results are shown in Table 1.

**Table 1:** The number of the infected, cured, deaths and removed

Date	Time point	Total infected	Cured	Death	Infected^1^	Removed^2^
2020.01.10	1	41	2	1	38	3
2020.01.11	2	41	6	1	34	7
2020.01.12	3	41	7	1	33	8
2020.01.13	4	41	7	1	33	8
2020.01.14	5	41	7	1	33	8
2020.01.15	6	41	12	2	27	14
2020.01.16	7	41	15	2	24	17
2020.01.17	8	62	19	2	41	21
2020.01.18	9	121	24	3	94	27
2020.01.19	10	198	25	3	170	28
2020.01.20	11	291	25	3	263	28
2020.01.21	12	440	28	9	403	37
2020.01.22	13	571	30	17	524	47
2020.01.23	14	830	34	25	771	59
2020.01.24	15	1287	38	41	1208	79
2020.01.25	16	1975	49	56	1870	105
2020.01.26	17	2744	51	80	2613	131
2020.01.27	18	4515	60	106	4349	166
2020.01.28	19	5974	103	132	5739	235
2020.01.29	20	7711	124	170	7417	294
2020.01.30	21	9692	171	213	9308	384
2020.01.31	22	11,791	243	259	11,289	502
2020.02.01	23	14,380	328	304	13,748	632
2020.02.02	24	17,205	475	361	16,369	836
2020.02.03	25	20,438	632	425	19,381	1057
2020.02.04	26	24,324	892	490	22,942	1382
2020.02.05	27	28,018	1153	563	26,302	1716
2020.02.06	28	31,161	1540	636	28,985	2176
2020.02.07	29	34,546	2050	722	31,774	2772
2020.02.08	30	37,198	2649	811	33,739	3460
2020.02.09	31	40,171	3281	908	35,982	4189
2020.02.10	32	42,638	3996	1016	37,626	5012
2020.02.11	33	44,653	4740	1113	38,800	5853
2020.02.12	34	59,804	5911	1367	52,526	7278
2020.02.13	35	63,851	6723	1380	55,748	8103
2020.02.14	36	66,492	8096	1523	56,873	9619
2020.02.15	37	68,500	9419	1665	57,416	11,084
2020.02.16	38	70,548	10,844	1770	57,934	12,614
**2020.02.17**	**39**	**72,436**	**12,552**	**1868**	**58,016**	**14,420**
2020.02.18	40	74,185	14,376	2004	57,805	16,380
2020.02.19	41	74,576	16,155	2118	56,303	18,273
2020.02.20	42	75,465	18,264	2236	54,965	20,500
2020.02.21	43	76,288	20,659	2345	53,284	23,004
2020.02.22	44	76,936	22,888	2442	51,606	25,330
2020.02.23	45	77,150	24,734	2592	49,824	27,326
2020.02.24	46	77,658	27,323	2663	47,672	29,986
2020.02.25	47	78,064	29,745	2715	45,604	32,460
2020.02.26	48	78,497	32,495	2744	43,258	35,239
2020.02.27	49	78,824	36,117	2788	39,919	38,905
2020.02.28	50	79,251	39,002	2835	37,414	41,837
2020.02.29	51	79,824	41,625	2870	35,329	44,495
2020.03.01	52	80,026	44,462	2912	32,652	47,374
2020.03.02	53	80,151	47,204	2943	30,004	50,147
2020.03.03	54	80,270	49,856	2981	27,433	52,837

^1^Infected = Total infected − cured − death; ^2^Removed = cured + death

### Estimation of model parameters

In order to get the value of the parameters in Eq. (3) based on data issued by NHCC, algorithm (*fmincon* and *lsqnonlin* function) was programmed to estimate the six parameters by matlab 2017b. In this study, all the parameters were defined to be nonnegative and bounded, because each parameter has its own significance. Secondly, based on real data, *fmincon* function was employed to estimate the approximate range of each parameter. Estimated parameters by the *fmincon* function were regarded as the initial values. Thereafter, a further estimation was performed using the *lsqnonlin* function to achieve the best fitting effect between the simulation curve and the real data curve.

### Average forecasting error rate

The equation of average forecasting error rate (AFER) is as follows:

4 $$ \textit{average error rate} = \frac{1}{n} \cdot\frac{\sum_{i = 1}^{n} \vert x_{i} - y_{i} \vert }{x_{i}} \times100\%, $$

where $x_{i}$ is the real value, $y_{i}$ is the forecasting value, *n* is the number of all data which need to be forecast.

## Results

### Parameters estimation and forecast

Based on Table 1, the method of data-driven modeling was adopted [27, 28]. In order to combat this epidemic, the Chinese government has adopted a series of measures, such as the establishment of Huoshenshan Hospital, Leishenshan Hospital, Fangcang Hospital, Wuhan quarantine, home isolation, and sending detachment of medical personnel. The simulation was divided into nine stages. The detailed steps are as follows.

*Stage 1*: from January 10, 2020 to January 21, 2020 (1th day to 12th day).

From January 10, 2020 to January 21, 2020, there were no quarantine measures. The initial values of the total population, the exposed, the infected, the removed, the death and the susceptible are $1.4 \times 10^{9}$, 0, 41, 2, 1 and ($1.4 \times10^{9} - 41 - 2$), respectively. The estimated average number of the infected contacting the susceptible per day (*r*) was 20, the estimated infection rate by the infected (*β*) was 0.043. Therefore, $\beta\cdot r = 0.86$ was similar to the reported literature [29]. The average number of the exposed contacting the susceptible per day ($r_{2}$) was 20, and because the probability of infection by the exposed is lower than that of the confirmed infected patients, we set $\beta_{2} = 0.025$. The probability that an exposed person turns into a confirmed infected patient (*δ*) was 0.079, which is similar to the reported literature [28]. The removal rate (*γ*) was 0.001, and is consistent with the reported literature [17]. The death rate ($\gamma_{0}$) was 0.0009.

*Stage 2*: from January 22, 2020 to February 3, 2020 (13th day to 25th day).

On January 22, 2020, Nanshan Zhong, a prominent Chinese physician led a panel of experts to Wuhan. Since he confirmed that the virus can spread from person to person, the government began to perform an isolation policy. However, because of the limited number of beds and isolation, *r* became 2 and $r_{2}$ became 7. At the same time, the government has sent some medical teams, and the removal rate (*γ*) has been improved to 0.0075. The death rate ($\gamma_{0}$) was 0.0045 and *δ* was 0.079. In the following stages, if not specified, the parameters are the same as the former stage. Measures have been taken on stage 2 as defined as intervention 1.

*Stage 3*: from February 4, 2020 to February 6, 2020 (26th day to 28th day).

Since February 4, 2020, as the government further improved the diagnosis efficiency and strengthened the isolation of communities, *r* was 0.1, $r_{2}$ was 2, *γ* was 0.02. $\gamma_{0}$ was 0.003. Measures have been taken on stage 3 as defined as intervention 2.

*Stage 4*: from February 7, 2020 to February 11, 2020 (29th day to 33th day).

From February 7, 2020 to February 11, 2020, the parameters were $r = 0.01$; $\beta = 0.0043$; $r_{2} = 1$; $\beta_{2} = 0.0025$; $\gamma = 0.025$; on February 12, 2020, $\delta = 0.05$, $\gamma = 0.03$, $\gamma_{0}$ was 0.0025.

*Stage 5*: from February 12, 2020 to February 14, 2020 (34th day to 36th day).

From February 12, 2020, diagnostic criteria have changed, and a positive nucleic acid test is not the only criterion. The government also added characteristic CT imaging patterns to the confirmed cases, so the number of infected cases was 52626 on February 12, 2020. Therefore, we revised the number of infected patients in our model. The parameters were $r = 0.01$; $r_{2} = 2$; $\gamma = 0.023$, $\delta = 0.12$, $\gamma_{0}$ was 0.0025.

*Stage 6*: from February 15, 2020 to February 17, 2020 (37th day to 39th day).

From February 15, 2020, to February 17, 2020, $r = 0.01$; $\beta = 0.0043$; $r_{2} = 1$; $\beta_{2} = 0.0025$; $\gamma = 0.03$; $\delta = 0.1$, $\gamma_{0}$ was 0.002.

*Stage 7*: February 18, 2020 to February 23, 2020 (40th to 45th).

From February 18, 2020, to February 23, 2020, $r = 0.01$; $\beta = 0.0043$; $r_{2} = 0.5$; $\beta_{2} = 0.0025$; $\gamma = 0.04$; $\delta = 0.04$, $\gamma_{0}$ was 0.002.

*Stage 8*: February 24, 2020 to February 28, 2020 (46th to 50th).

From February 24, 2020 to February 28, 2020, $r = 0.01$; $\beta = 0.0043$; $r_{2} = 0.1$; $\beta_{2} = 0.0025$; $\gamma = 0.07$; $\delta = 0.04$, $\gamma_{0}$ was 0.001.

*Stage 9*: February 29, 2020 to March 3, 2020 (51th to 54th).

From February 29, 2020 to March 3, 2020, $r = 0.01$; $\beta = 0.0043$; $r_{2} = 0.1$; $\beta_{2} = 0.0025$; $\gamma = 0.085$; $\delta = 0.02$, $\gamma_{0}$ was 0.001.

From March 4, 2020, with the increase of removal rate and greater isolation strength, the parameter *γ* will be larger, $\gamma_{0}$, *r* and $r_{2}$ will be smaller.

### Correlation and average forecasting error rate

Based on the aforementioned nine stages, the date January 10, 2020 was regarded as the starting point, and the data from January 10 to February 15, 2020 was regarded as the training set for model parameter estimation. The $R_{I}$ between established model data in this study and issued data by NHCC is 99.9% and the $R_{R}$, $R_{D}$ and $R_{C}$ were 99.8%, 99.8% and 99.6%. The data from February 16, 2020 to March 3, 2020 were used to forecast and verify the model. The data of the number of the infected of model forecast and issued by NHCC were shown in Table 2. Therefore, the AFER of the infected was 0.78%. Similarly, the AFER of the removed, the death and the cured were 0.75%, 0.35% and 0.83%, respectively (see Table 2).

**Table 2:** The forecasting infected and removed from February 16 to March 3

Day	Infected	Forecasting Infected	Removed	Forecasting Removed	Death	Forecasting Death	Cured	Forecasting Cured
38	57,934	58,217	12,614	12,684	1770	1796	10,844	10,907
39	58,016	58,338	14,420	14,431	1868	1912	12,552	12,537
40	57,805	58,274	16,380	16,181	2004	2029	14,376	14,171
41	56,303	56,552	18,273	18,512	2118	2146	16,155	16,385
42	54,965	54,875	20,500	20,774	2236	2259	18,264	18,534
43	53,284	53,242	23,004	22,969	2345	2369	20,659	20,619
44	51,606	51,654	25,330	25,099	2442	2475	22,888	22,643
45	49,824	50,107	27,326	27,165	2592	2578	24,734	24,606
46	47,672	48,603	29,986	29,169	2663	2679	27,323	26,510
47	45,604	45,682	32,460	32,572	2715	2727	29,745	29,863
48	43,258	42,945	35,239	35,769	2744	2773	32,495	33,015
49	39,919	40,383	38,905	38,775	2788	2816	36,117	35,978
50	37,414	37,982	41,837	41,602	2835	2856	39,002	38,765
51	35,329	35,732	44,495	44,261	2870	2894	41,625	41,386
52	32,652	32,891	47,374	47,298	2912	2930	44,462	44,387
53	30,004	30,288	50,147	50,094	2943	2962	47,204	47,150
54	27,433	27,902	52,837	52,668	2981	2993	49,856	49,694

### The dynamic trends of the susceptible, the exposed, the infected and the removed

**Figure 2:**
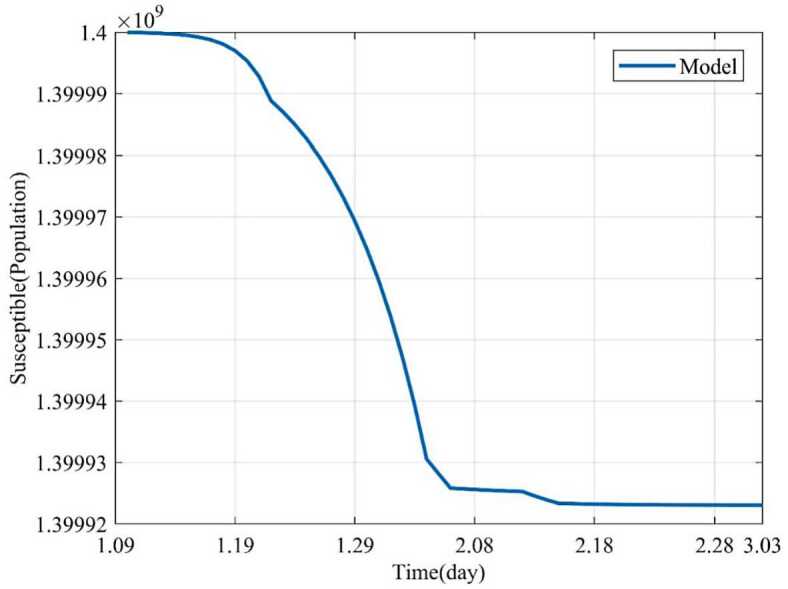
Dynamic trends of the susceptible

**Figure 3:**
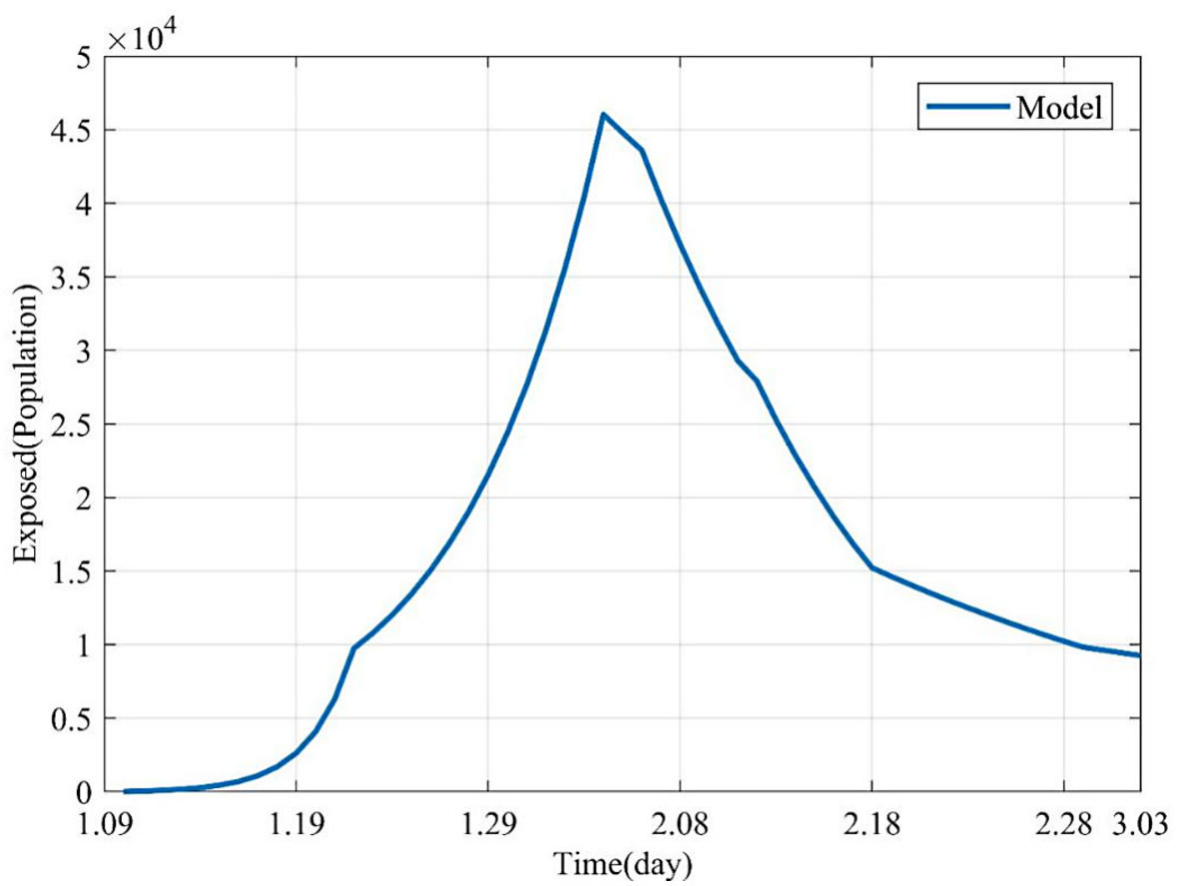
Dynamic trends of the exposed

**Figure 4:**
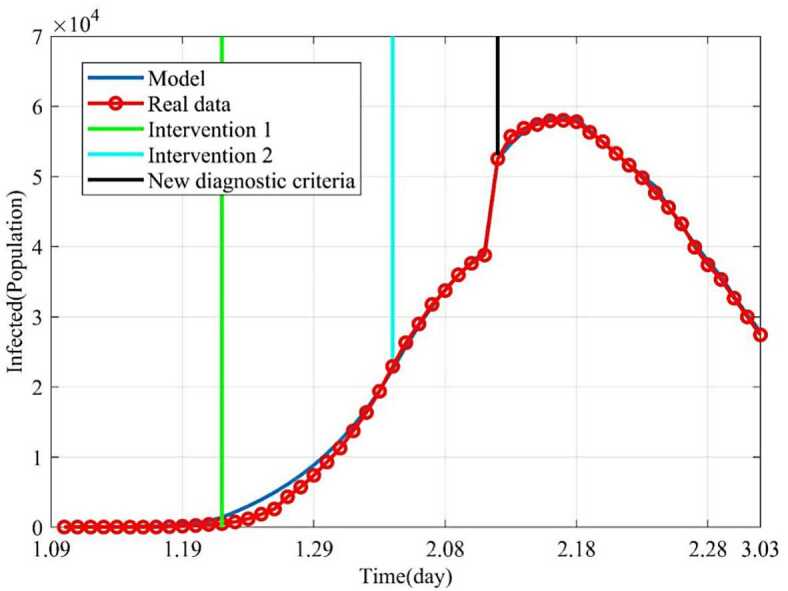
Dynamic trends of the infected. The blue line represents the simulation of SEIR model. The red line represents the data issued by NHCC. The green line represents the intervention 1. The light blue line represents the intervention 2. The black line represents that the government began to implement new diagnostic criteria

**Figure 5:**
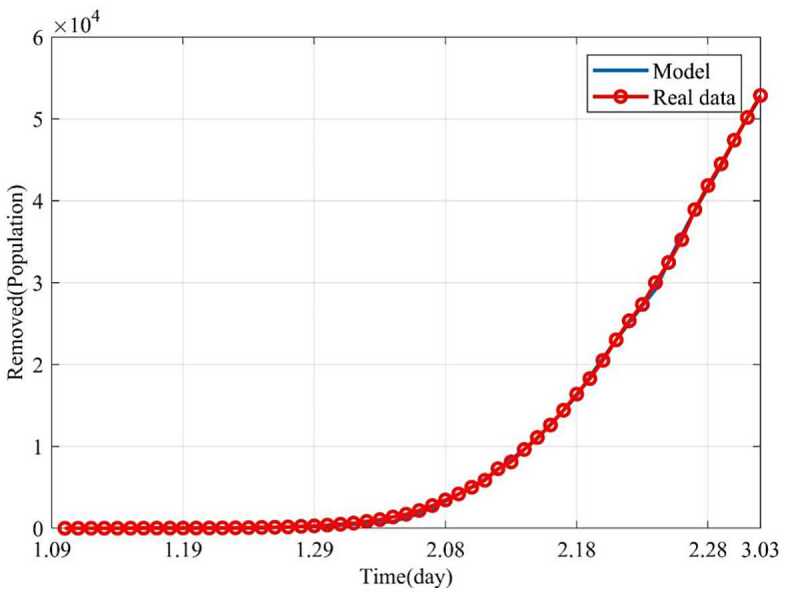
Dynamic trends of the removed. The removed consist of the cures and the death. The blue line represents the simulation of SEIR model. The red line represents the data issued by NHCC

**Figure 6:**
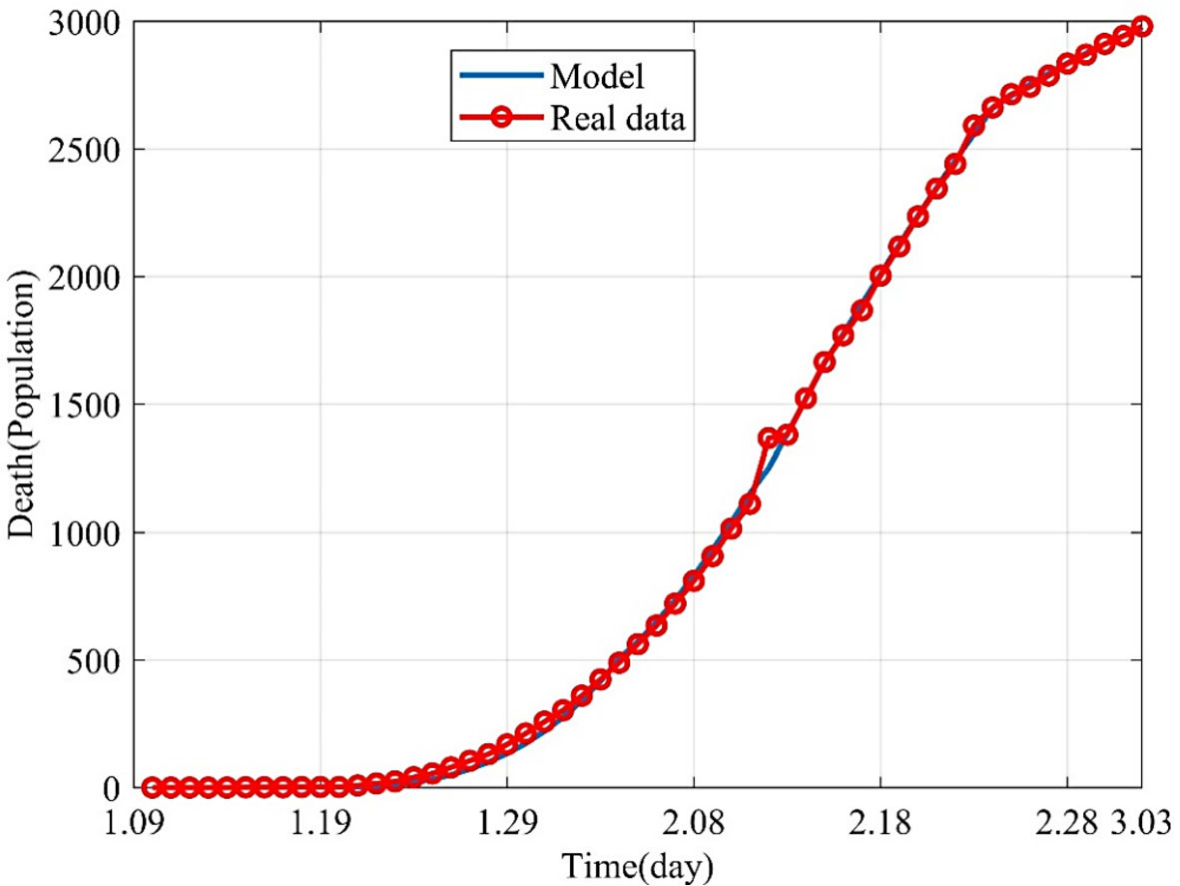
Dynamic trends of the death. The blue line represents the simulation of SEIR model, and the red line represents the data issued by NHCC

**Figure 7:**
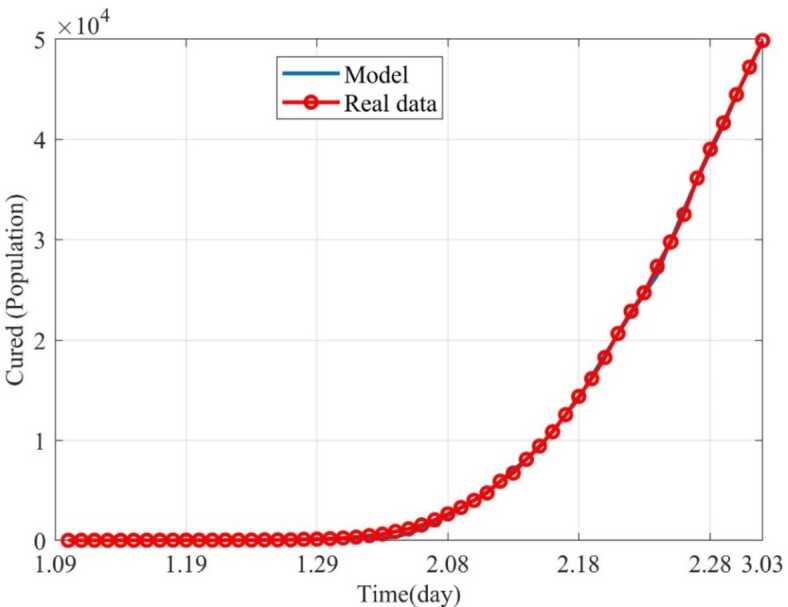
Dynamic trends of the cured. The blue line represents the simulation of SEIR model, and the red line represents the data issued by NHCC

Based on the above nine stages, the dynamic trends of the susceptible, exposed, infected and removed for 54 days (from January 10, 2020 to March 3, 2020) were simulated. Figure 2 showed the dynamic trends of the susceptible where the initial population is ($1.4 \times10^{9} - 41 - 2$) and has a sharp drop from January 19, 2020, to February 5 and remain relatively stable. The dynamic trends of the exposed showed the number of the exposed increased consistently and reached its peak on February 4 and began to gradually decrease (see Fig. 3). The dynamic trends of the infected showed that, when the government took intervention 2, the growth rate of the infected population decreased significantly. When diagnostic criteria have changed, namely, the government added characteristic CT imaging patterns to the confirmed cases, there was a sudden increase of the number of the infected. The number of the infected reached the peak on February 17 and gradually began to decrease (see Fig. 4). The dynamic trends of the removed showed that the number of the removed continued to increase which is consistent with the data issued by NHCC (see Fig. 5). The number of the death increased quickly from January 10 to February 23 and then slowly from February 24 to March 03 (see Fig. 6). At the beginning, the number of the cured increased slowly, however, from February 08, more and more patients were cured (see Fig. 7).

### Influence of parameters on epidemic

#### Influence of removal rate on epidemic (*γ*)

**Figure 8:**
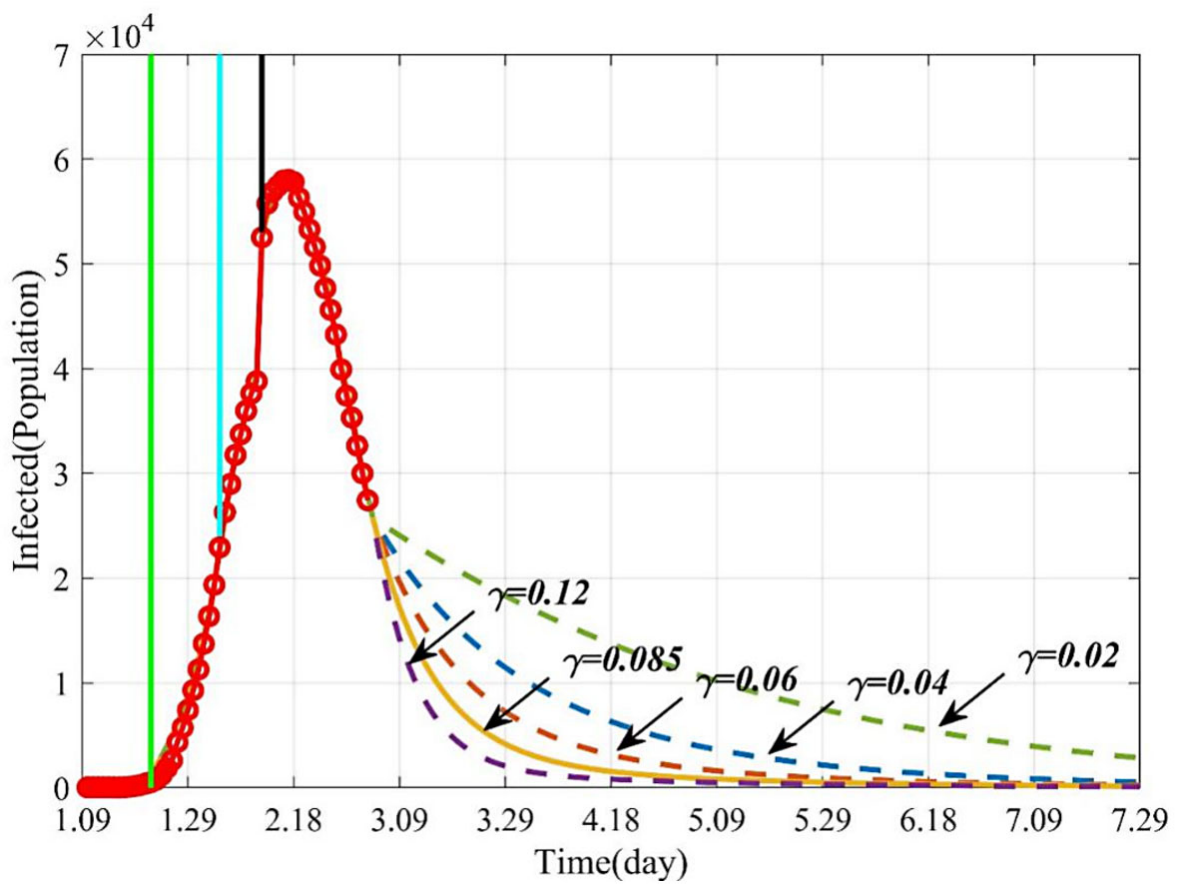
Dynamic trends of the infected with different removal rates (*γ*). The solid line represents the trends of the infected with the current *γ*. The purple dotted line represents the trends of the infected when $\gamma = 0.12$. The orange dotted line represents the trends of the infected when $\gamma = 0.06$. The blue dotted line represents the trends of the infected when $\gamma = 0.04$. The green dotted line represents the trends of the infected when $\gamma = 0.02$

**Figure 9:**
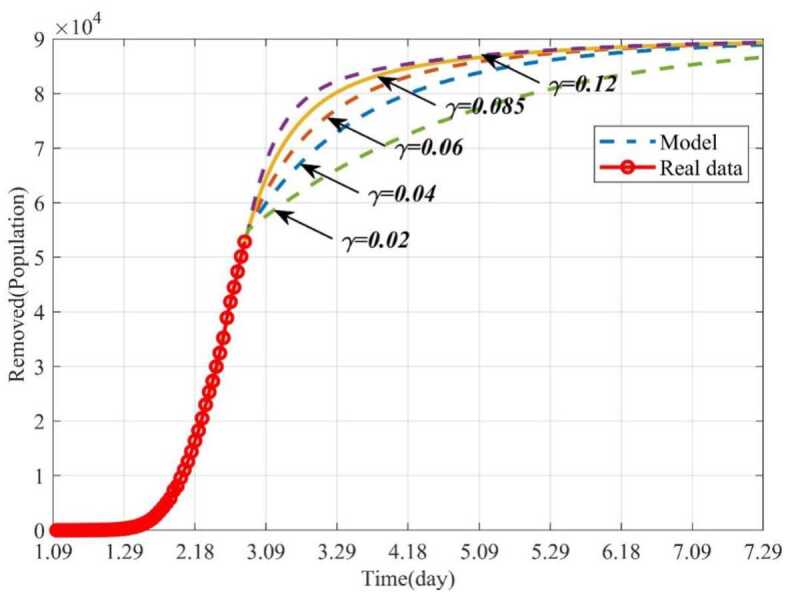
Dynamic trends of the removed with different removal rates (*γ*). The solid line represents the trends of the removed with the current *γ*. The purple dotted line represents the trends of the removed when $\gamma= 0.12$. The orange dotted line represents the trends of the removed when $\gamma= 0.06$. The blue dotted line represents the trends of the removed when $\gamma= 0.04$. The green dotted line represents the trends of the removed when $\gamma= 0.02$

Because the removed consisted of the death and the cured, we just analyzed the Influence of removal rate on epidemic. From the 55th day (March 4, 2020), the influence of the removal rate (*γ*) on the infected and the removed were investigated on condition of changing the removal rate (*γ*) and fixing other parameters. *γ* was gradually increased from 0.02 to 0.12. As shown in Fig. 8, the dynamic trends of the infected with different removal rates suggested the larger the removal rate is, the better the control effect is. When $\gamma = 0.02$, 200 days later (July 29), there are still large numbers of infected individuals, while for $\gamma = 0.12$, 135 days later (May 25), there will be no infected individuals (see Fig. 8). Similarly, if $\gamma = 0.02$, 200 days later (July 29), the number of the removed individuals increases, namely, there are still many patients that need to be treated. If $\gamma = 0.12$, the number of the removed becomes stable (Fig. 9). Therefore, it indicates that if the removal rate can be improved, the epidemic will be effectively controlled and terminated earlier. From Fig. 9, we also get the conclusion that the larger the removal rate is, the shorter the time it takes for the removed to become stable. This also corresponds to the shorter time to reach the stable point in Fig. 8. Since the government sent a large number of medical teams to support Wuhan, the epidemic was effectively controlled and the number of the infected decreased, which is consistent with our forecasting results.

#### Influence of the average number of the infected contacting the susceptible per day (*r*) on the epidemic

**Figure 10:**
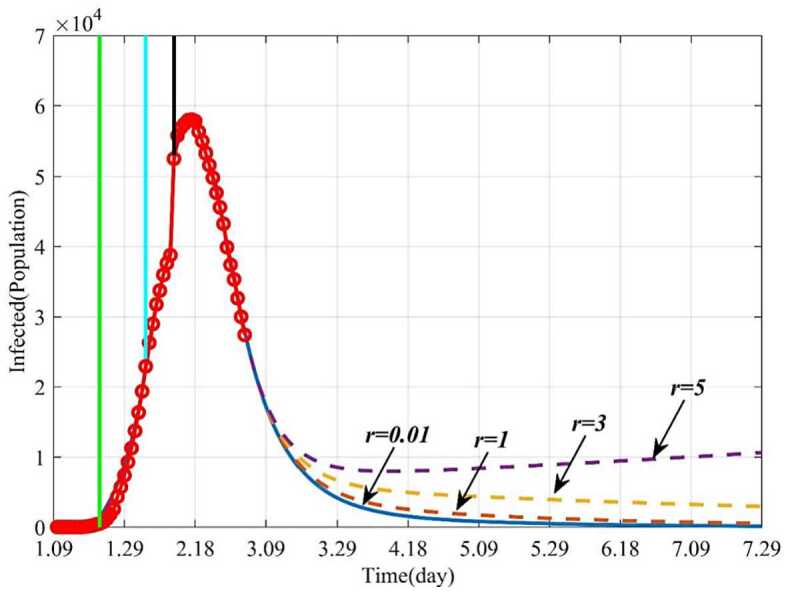
Dynamic trends of the infected with different *r*. The solid line represents the trends of the infected with the current *r*. The orange dotted line represents the trends of the infected when $r = 1$. The yellow dotted line represents the trends of the infected when $r = 3$. The purple dotted line represents the trends of the infected when $r = 5$

**Figure 11:**
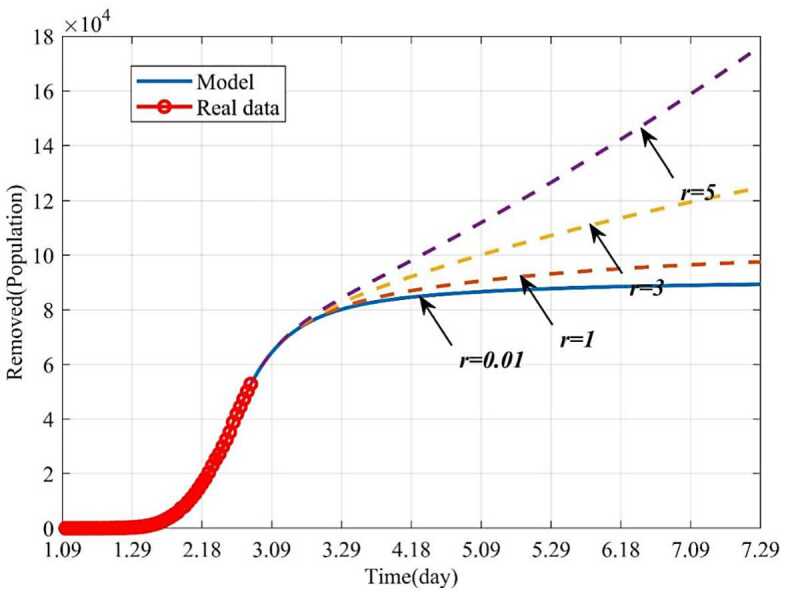
Dynamic trends of the removed with different *r*. The solid line represents the trends of the removed with the current *r*. The orange dotted line represents the trends of the removed when $r = 1$. The yellow dotted line represents the trends of the removed when $r = 3$. The purple dotted line represents the trends of the removed when $r = 5$

This section studied how the average number of the infected contacting the susceptible per day (*r*) influences the infected and the removed when other parameters keep fixed. The epidemic can be well controlled when $r = 0.01$, and finally the infected individuals will disappear. But if the parameter *r* gradually increases from 0.01 to 5, the number of the infected will increase, and the epidemic will be out of control (Fig. 10). Figure 11 showed trends of the removed with different *r*. If $r = 0.01$, 160 days later (June 18), the removed will not increase and remain stable. But if the isolation rate $r \ge3$, it will take a long time for the epidemic to be effectively controlled, or even out of control. For example, if $r = 5$, the number of the infected will increase continually. This is because more persons will be infected and need to be treated. Therefore, timely isolation of the infected and close contacts, and establishment of Huoshenshan Hospital, Leishenshan Hospital and Fangcang Hospital will be very effective.

#### Influence of the average number of the exposed contacting the susceptible per day ($r_{2}$) on the epidemic

**Figure 12:**
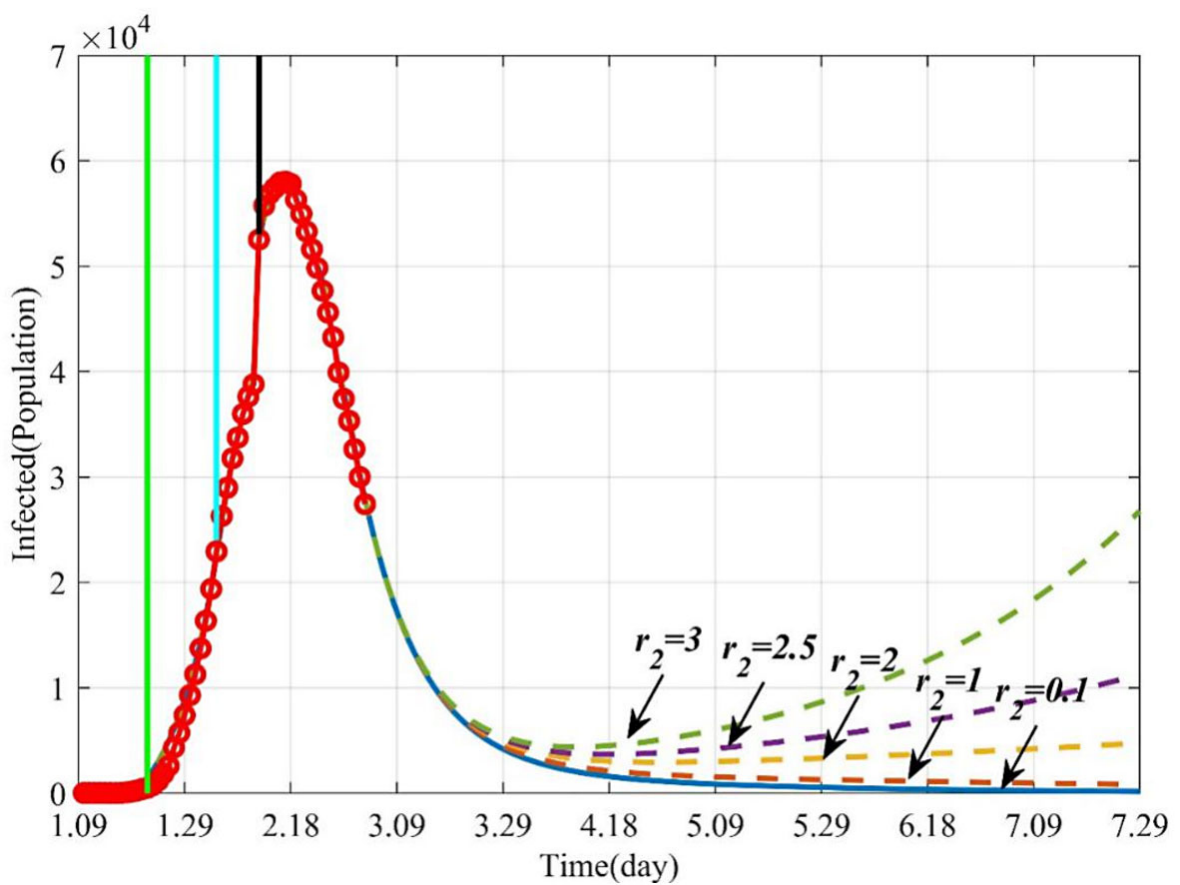
Dynamic trends of the infected with different $r_{2}$. The solid line represents the trends of the infected with the current $r_{2}$. The orange dotted line represents the trends of the infected when $r_{2} = 1$. The yellow dotted line represents the trends of the infected when $r_{2} = 2$. The purple dotted line represents the trends of the infected when $r_{2} = 2.5$. The green dotted line represents the trends of the infected when $r_{2} = 3$

**Figure 13:**
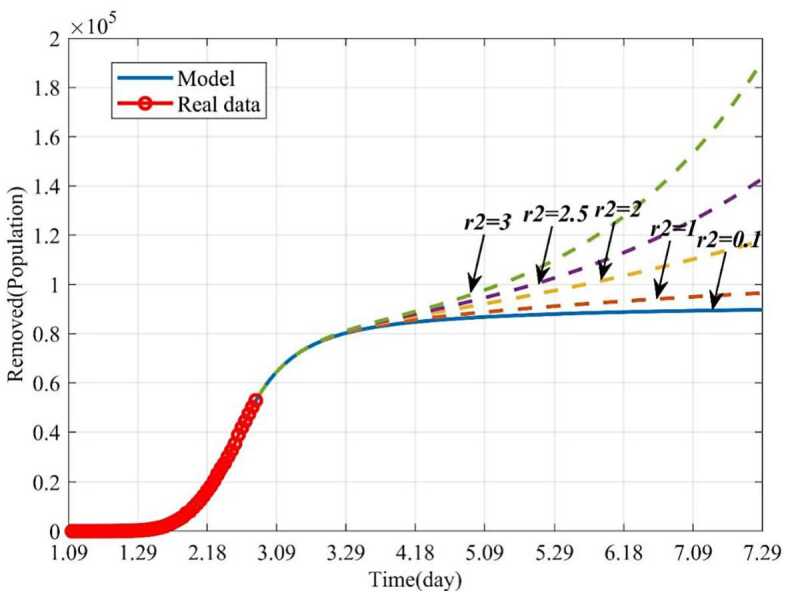
Dynamic trends of the removed with different $r_{2}$. The solid line represents the trends of the removed with the current $r_{2}$. The orange dotted line represents the trends of the removed when $r_{2} = 1$. The yellow dotted line represents the trends of the removed when $r_{2} = 2$. The purple dotted line represents the trends of the removed when $r_{2} = 2.5$. The green dotted line represents the trends of the removed when $r_{2} = 3$

In this section, the influence of the average number of the exposed contacting the susceptible per day ($r_{2}$) on the epidemic was studied by numerical simulation. When $r_{2} = 0.1$ and other parameters keep fixed, 160 days later (June 18), the infected will disappear. If the parameter $r_{2}$ increases from 0.1 to 3, the epidemic will become uncontrollable. For example, when $r_{2} = 3$, the number of infections will decrease on the beginning and then increase gradually, and the epidemic will outbreak again (see Fig. 12). Similarly, more patients will need to be treated (see Fig. 13).

## Discussion

Due to the rapid spread of COVID-19 and no vaccine or effective treatment being available for the epidemic, it has been declared by the World Health Organization as an “international public health emergency”. Even though the Chinese government has exerted massive efforts to fight against the epidemic COVID-19, there are still new cases of infection every day. In order to scientifically forecast the spread tendency of the disease, we established a mathematical model on COVID-19. The forecasting accuracy of our model has been confirmed by the data issued by NHCC.

In this study, we first modified a classical SEIR model and established a mathematical model according to the possible transmission mechanism of COVID-19. Based on the official data issued by NHCC, the model parameters were estimated and the trends of the infected and the removed populations were forecast in the short term. The average forecasting error rates of the infected and the moved were 0.78% and 0.75%, respectively. Next, series of parameters on this epidemic were studied, including the influences of removal rate (*γ*), the average number of the infected contacting the susceptible per day (*r*) and the average number of the exposed contacting the susceptible per day ($r_{2}$). Compared with the reported mathematical models, our model is more effective and accurate, for example, Natsuko et al. [30] forecast there will be 4000 infected in China, however, the NHCC issued the number of infections is 94 on January 18, 2020. Gardner et al. [31] predicted that, as of January 25, 2020, there will be 20,000 people infected, and the number of infections issued by the NHCC is 1870. The SEIR model established by Ai et al. [29] predicted that the peak of the epidemic in Hubei province will be between January 28 and February 7, 2020, and the number of infected people will be 7000 to 9000, in fact, the peak time of the epidemic is February 18. However, the main advantages of our model are that the correlation of the infected ($R_{I}$) between model data and issued data by NHCC is as high as 99.9%, the correlations of the removed ($R_{R}$), the death ($R_{D}$) and the cured ($R_{C}$) are as high as 99.8%, 99.8% and 99.6%, respectively. Besides, the AFER of the infected, the removed, the death and the cured are as low as 0.78%, 0.75%, 0.35% and 0.83%, respectively. Therefore, the forecast data derived from our model are approximately validated by the real data issued by NHCC

We further explored how the parameters influence the epidemic by numerical simulations. The results showed that when increase the removal rate (*γ*) and keep other parameters fixed, the time to control the epidemic will be greatly shortened. For example, if increase the removal rate (*γ*) from 0.02 to 0.12 and keep other parameters fixed, the disappeared time of the epidemic will be advanced from July 29 to May 25. This theoretical induction has been proved as the number of the cured increased, since government dispatched medical teams to Hubei province.

Besides, if the average number of the infected contacting the susceptible per day (*r*) can be effectively reduced, the epidemic can be controlled earlier. For instance, if *r* is reduced from 5 to 0.01, the epidemic will disappear by May 25, and if $r = 5$, the number the infected will be increasing. In addition, if the average number of the exposed contacting the susceptible per day $r_{2}$ is 3, the epidemic will break out again, and if $r_{2}$ is controlled to be less than 0.1, the epidemic will terminate soon. These results have been proved as the number of the infected reached the peak and then decreased, since government has demonstrated an unprecedented level of efforts in dealing with the COVID-19, such as to set up specialized hospitals for nCoV patients, namely Huoshenshan and Leishenshan hospital, Fangcang Hospital.

In conclusion, our established mathematical model can provide theoretical guidance for effective prevention and control of the epidemic COVID-19 in China. With appropriate modifications, it could be applied for other countries currently attacked by the epidemic.
